# Monitoring bone changes due to calcium, magnesium, and phosphorus loss in rat femurs using Quantitative Ultrasound

**DOI:** 10.1038/s41598-018-30327-7

**Published:** 2018-08-10

**Authors:** Aldo Fontes-Pereira, Paulo Rosa, Thiago Barboza, Daniel Matusin, Aline Soares Freire, Bernardo Ferreira Braz, Christiano Bittencourt Machado, Marco Antônio von Krüger, Sergio Augusto Lopes de Souza, Ricardo Erthal Santelli, Wagner Coelho de Albuquerque Pereira

**Affiliations:** 1 Ultrasound Laboratory, Biomedical Engineering Program/COPPE/Federal University of Rio de Janeiro - UFRJ, Cidade Universitária, Centro de Tecnologia, Bloco H, PO Box 68510, Rio de Janeiro, RJ 21945-970 Brazil; 2 Nuclear Medicine Service, Clementino Fraga Filho University Hospital, Cidade Universitária, Rio de Janeiro, RJ 21941-913 Brazil; 3 Departamento de Química Analítica, Av. Athos da Silveira Ramos, 149 - Centro de Tecnologia Federal, University of Rio de Janeiro, Cidade Universitária, Rio de Janeiro, RJ 24020-007 Brazil; 4 Biomedical Ultrasound Laboratory, Estácio de Sá University, Rio de Janeiro, RJ 20261-063 Brazil

## Abstract

Bone mineral density is an important parameter for the diagnosis of bone diseases, as well as for predicting fractures and treatment monitoring. Thus, the aim of the present study was to evaluate the potential of Quantitative Ultrasound (QUS) to monitor bone changes after calcium, phosphorus, and magnesium loss in rat femurs *in vitro* during a demineralization process. Four quantitative ultrasound parameters were estimated from bone surface echoes in eight femur diaphysis of rats. The echo signals were acquired during a decalcification process by Ethylenediaminetetraacetic Acid (EDTA). The results were compared to Quantitative Computed Tomography (QCT) and inductively coupled plasma optical emission spectrometry measurements for validation. Integrated Reflection Coefficient (IRC) reflection parameters and Frequency Slope of Reflection Transfer Function (FSRTF) during demineralization tended to decrease, while the backscattering parameter Apparent Integrated Backscatter (AIB) increased and Frequency Slope of Apparent Backscatter (FSAB) showed an oscillatory behavior with no defined trend. Results indicate a clear relation between demineralization and the corresponding decrease in the reflection parameters and increase in the scattering parameters. The trend analysis of the fall curve of the chemical elements showed a better relationship between IRC and QCT. It was possible to monitor bone changes after ions losses, through the QUS. Thus, it is an indication that the proposed protocol has potential to characterize bone tissue in animal models, providing consistent results towards standardization of bone characterization studies by QUS endorsing its use in humans.

## Introduction

According to the World Health Organization, osteoporosis is responsible for more than 8.9 million fractures annually worldwide, of which approximately 4.5 million cases occur in America and Europe^1^. It is the most common osteometabolic disease among the elderly, posing great challenges for contemporary public health^2–4^. For example, it is estimated that the lifetime risk for a wrist, hip, or vertebral fracture is up to 40% in developed countries, which is nearly equal to the lifetime risk for coronary heart disease^1^. Another important fact is that the costs of osteoporotic fracture treatment are high^5,6^. In the United States, there are more than 3 million fractures per year, and the cost of $25 billion is predicted for 2025^6^.

Most studies on osteoporosis stress the importance of prevention. Adequate calcium and vitamin D intake, regular physical activity, strategies to prevent falls, and avoiding addictions are among the common recommendations. Approved therapies encompass bisphosphonates, Selective Estrogen Receptor Modulators (SERMs), estrogen, calcitonin, parathyroid hormone, the Receptor Activator of Nuclear Factor-Kappa B Ligand (RANKL) inhibitor, denosumab, and strontium ranelate^7^.

It is well accepted that Bone Mineral Density (BMD [g/cm^3^]) is the main parameter for the diagnosis of osteoporosis, as well as for predicting fractures and monitoring treatment. Dual-energy X-ray Absorptiometry (DXA [g/cm^2^]) and Quantitative Computed Tomography (QCT [g/cm^3^]) are the two most common ionizing techniques to estimate BMD. Although QCT is more sensitive than DXA, QCT imposes high costs and imposes higher exposures to radiation to acquire an adequate image^8,9^. On the other hand, even when clinical assessment indicates osteoporosis, DXA may suggest a normal BMD^10^. According to Seo *et al*.^11^, several investigators have been focusing on the accuracy of BMD in predicting bone status by evaluating QCT and DXA values, and a few studies estimated the correlation between bone biomechanical properties and BMD with the same purpose.

An attractive alternative to ionizing diagnostic techniques is Quantitative Ultrasound (QUS), which has the advantages of low-cost, easily transportable devices and being free of ionizing radiation^12^. QUS refers to a set of possible parameters extracted from the transmitted or backscattered Ultrasound Radiofrequency (RF) signals after its propagation through the biological tissue. Examples of QUS parameters are the Speed of Sound (SOS)^13^, Broadband Ultrasound Attenuation (BUA) coefficient^14^, backscattering coefficient^15^, and Mean Scatterer Spacing (MSS)^16^. Thus far, QUS has demonstrated potential in predicting osteoporotic fractures in postmenopausal women and men over 65 years old^17^. However, great challenges remain with respect to treatment initiation, treatment monitoring, and quality control^18^. Several studies^13,14,19,20^ have used ultrasonic parameters, mainly BUA or SOS, with varying degrees of success to characterize bone. However, bone tissue cannot be adequately characterized by one or two parameters obtained in only a few skeletal sites. Studies are ongoing to develop new measurement modes and model-based signal-processing techniques^19^.

Three important bone minerals are calcium (Ca), magnesium (Mg), and phosphorus (P). The major mineral constituent of bone is calcium phosphate in the form of hydroxyapatite^21^. Magnesium also plays a key role in bone and mineral homeostasis since it affects the formation/secretion of hormones that regulate skeletal homeostasis and bone cell function, and influences hydroxyapatite crystal formation and growth. People with poor Mg intake may have hypocalcemia because of deficient Parathyroid Hormone (PTH) secretion and PTH end-organ resistance, as well as a higher risk for osteoporosis^22^. On the other hand, phosphorus is essential in cellular physiology and skeletal mineralization since (a) it participates on the composition of nucleic acids, hydroxyapatite, and adenosine triphosphate (ATP); (b) it forms phospholipids in cell membranes; (c) it participates on a variety of enzymatic reactions (e.g., glycolysis) and protein functions (e.g., hemoglobin oxygen transport). Hypophosphatemia diminishes osteoid mineralization and accounts for the ensuing rickets and/or osteomalacia^23^.

Several decalcification protocols have been proposed previously^24–26^, some of which assess the effect of demineralization with QUS parameters^27^. However, they did not take into account the presence of magnesium in bone samples. As BMD influences ultrasonic bone characterization and there is no standardization of measurement protocol, which may limit applications in clinical trials, further research is needed to quantify mineral content and QUS parameters with more confidence.

The aim of the present study was to evaluate the potential of QUS, especially reflection and backscattering parameters, to monitor bone changes after calcium, phosphorus, and magnesium loss in rat femurs *in vitro* during a bone demineralization process. The results are compared to QCT and inductively coupled plasma optical emission spectrometry (ICP OES) measurements. The choice of rat bones is justified by the fact that, with the exception of bones of other primates, rat bones are most similar to human bones from a histomorphological perspective^28,29^. Furthermore, rats are frequently used as animal models for the evaluation of metabolic bone diseases and pathophysiological conditions^29,30^.

## Results

**Figure 1:**
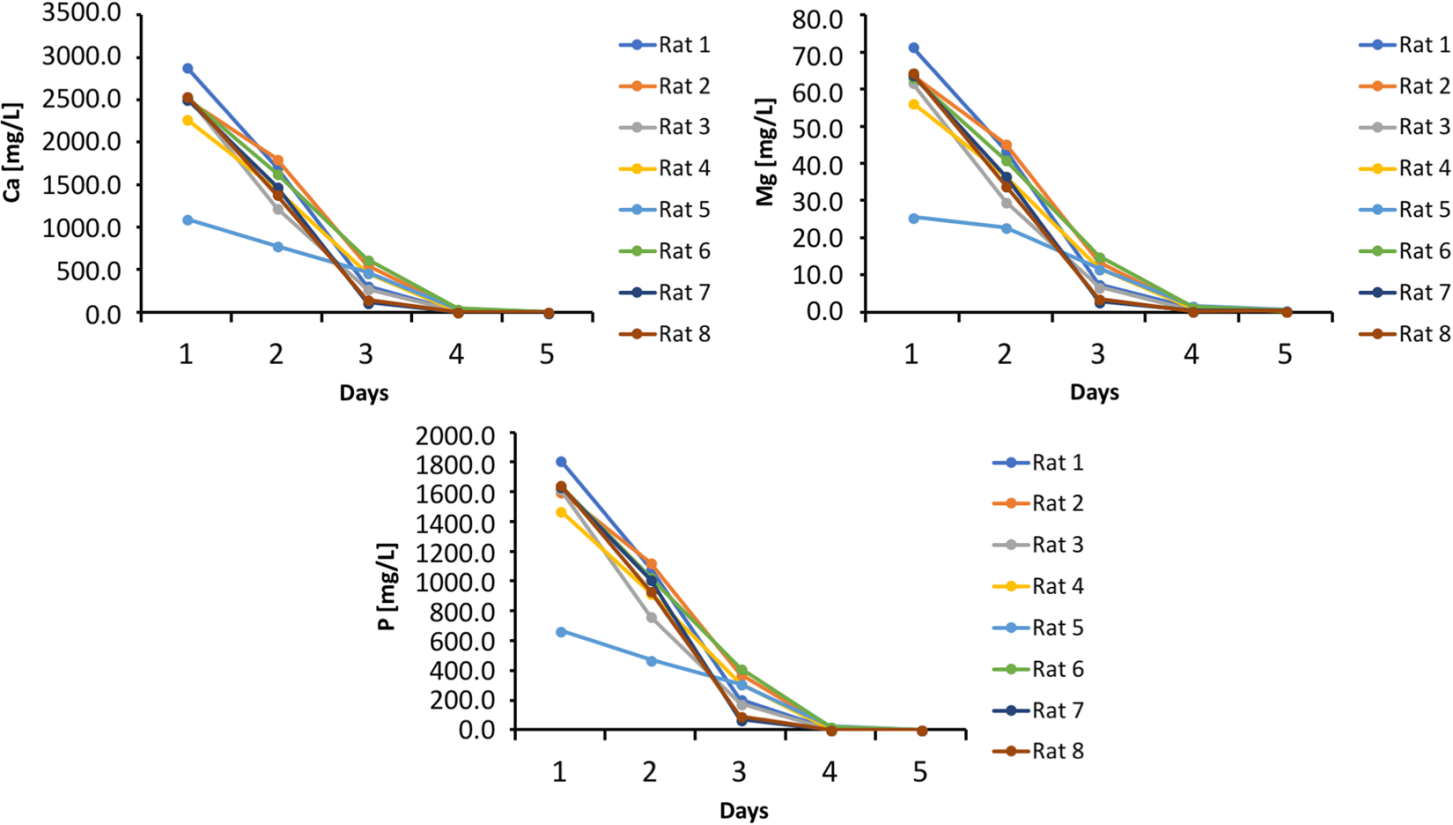
Variation of Ca, Mg, and P concentrations in EDTA solutions during the process of demineralization.

The concentrations of Ca, Mg, and P in the EDTA solutions during the period of demineralization indicate a rapid mineral loss in the first four days, reaching a plateau after this point. Figure 1 shows the variation of elements (Ca, Mg, and P) in the EDTA solution during demineralization. The correlations between the elements were very strong and positive (r = 0.99; p ≤ 0.0001).

**Figure 2:**
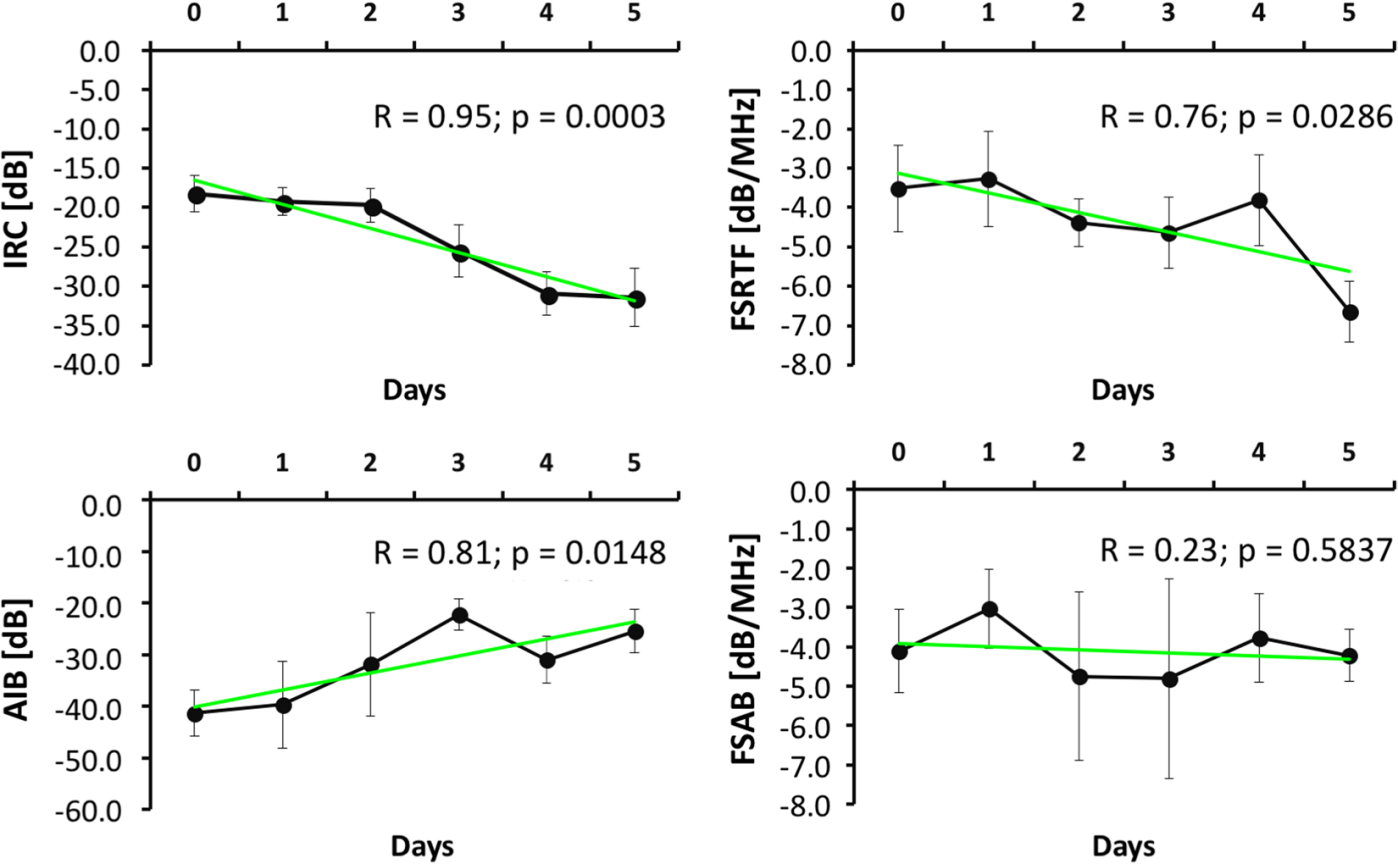
Trend of the four ultrasound parameters of the samples throughout the demineralization process. R: Linear regression.

The samples showed similar trends in the 4 parameters throughout the demineralization process. The IRC reflection parameters and FSRTF during demineralization showed a trend of decrease (IRC: R = 0.95; p = 0.0003, FSRTF: R = 0.76; p = 0.0286), while the backscattering parameter AIB increased (R = 0.81; p = 0.0148) and FSAB showed an oscillatory behavior with no defined trend (R = 0.23; p = 0.5837), as shown in Fig. 2.

**Figure 3:**
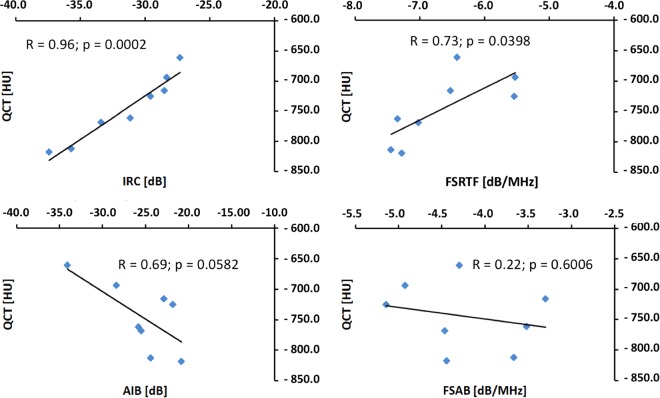
Correlation between each QUS parameter and QCT for the last day for each sample. R: Coefficient of correlation.

The correlation between each QUS parameter and QCT for the last day for each sample is shown in Fig. 3.

## Discussion

To our knowledge, this work is the first to propose an *in vitro* mineral loss monitoring methodology using QUS parameters and QCT together, and it constitutes an important contribution to ultrasound as an adjuvant tool for the diagnosis and monitoring of bone healing and bone disease. Thus, this is an important step toward preclinical trials and subsequent application of QUS in several diseases, such as osteoporosis^20^, osteoarthritis^31^, osteomyelitis^32^, and Paget’s disease^31^. Several methods exist to diagnose injuries and bone diseases, such as DXA and QCT. The advantages of QUS in relation to QCT include the low operating costs, easy equipment handling, and exposition to nonionizing radiation. Moreover, QUS can minimize subjectivity in diagnosis or monitoring made by conventional X-rays.

Previous studies showed that there is a higher mineralization degree in trabecular bone of osteoporotic individuals, with a low BMD, indicating that individuals with osteoporosis can still present a higher mineral content despite bone loss. The collagen matrix may also be influenced showing a functional deficiency due to increased intermolecular cross-linking^33^. However, it can be considered that the present approach is an alternative to better understand the relation between mineral concentrations in bone inorganic matrix and diagnostic parameters from ultrasound and ionizing techniques.

Several studies have been performed for the diagnostics of bone injury and for monitoring bone healing, including mathematical simulations^34^, *in vitro* studies^27^ and animal model studies^35^ with different degrees of success. However, the results of those studies cannot be extrapolated to humans for many reasons, mainly the lack of reproducibility. Machado *et al*.^27^ introduced a significant contribution for the advancement of QUS bone characterization by using one bovine bone with increasing degrees of decalcification by EDTA and analyzing the relationship of the QUS parameters with the ratio of the amount of calcium loss in the bone. Thus, it was expected that we can achieve further advancement in this direction by performing monitoring in eight bones of rats using four ultrasonic parameters. In the present study, a demineralization protocol similar to that proposed by Machado *et al*.^27^ was applied in rat bones for the first time with a larger number of samples. Rats are interesting animal models for the evaluation of metabolic bone diseases and certain pathophysiological conditions^29,30^, and their bones are similar to human bones^28,29^. We used four potential QUS parameters (especially reflection and backscattering parameters), together with an analysis of chemical elements related to the density and bone quality, i.e. calcium, phosphorus, and magnesium, and computed tomography images.

The lateral width of the 5-MHz US beam was smaller than the bone thickness. Ultrasonic signals were evaluated at three points along the bone diaphysis controlled by a stereotaxic holder, guaranteeing evaluation within the ROI, a signal with lower influences of the anatomical variables, and better repeatability^12^.

There is a consensus that BMD is a major parameter for the diagnosis of osteoporosis, as well as for the prediction of fractures and bone disease and the monitoring of treatments. DXA and QCT are two ionizing techniques for estimating BMD. QCT is more sensitive than DXA, but it is 2 to 3 times more expensive and requires higher levels of radiation to obtain better accuracy^8,9^. In addition, even when clinical evaluation indicates osteoporosis, in some cases, DXA is not sensitive and does not detect osteoporosis^10^. According to Seo *et al*.^11^, it is pertinent to discuss the accuracy and differences of values for detecting changes in BMD, QCT, and DXA.

The main bone mineral is calcium phosphate in the form of hydroxyapatite crystals. Magnesium also plays a key role in good mineral balance since it is directly related to the formation and secretion of hormones that regulate skeletal homeostasis and bone cell functions, and it acts on the growth and formation of hydroxyapatite crystals. Several methods for bone demineralization have been explored previously^24,26^, some of which were used to evaluate the effects of demineralization on QUS parameters^27^.

In the analysis of bone minerals, the decay curves of Ca, Mg, and P were correlated to each other, even for rat 5, which did not have epiphysis. This correlation was confirmed by the fact that the correlations among Ca, Mg, and P were positive and very strong. The inorganic matrix is formed predominantly by Ca and P in the form of hydroxyapatite crystals, which are responsible for the properties of stiffness and resistance to compression. Thus, the proportional extraction of these two ions suggests that EDTA demineralization occurred in such a manner that it altered bone quality as well as bone density. The present EDTA demineralization protocol has potential for studies of QUS parameters for clinical purposes. In addition, the strong correlation between the elements confirms the studies that show the relationship of Mg intake with bone quality. Mg is a regulator of calcium that controls calcium metabolism for the maintenance of blood homeostasis and bone matrix formation. In addition, it participates in the activation of vitamin D, which is important for the absorption of calcium by the bones. Rude *et al*.^22^ suggested that a low Mg concentration can compromise the secretion of the parathyroid hormone (PTH) affecting hypocalcemia and even osteoporosis. The strong correlation among the elements reinforces the need for analysis of elements other than Ca (Mg and P, for example) in studies of bone characterization by QUS. This strong correlation suggests that the individual behavior of each ion loss versus the QUS parameters will be similar to the ones presented in Fig. 2. Solely the dosage of calcium in bone does not guarantee good bone quality analysis.

As a general rule, the four parameters presented a moderate correlation with the amount of minerals extracted by EDTA. This result suggests that the ultrasonic parameters, especially the IRC, have the potential to be used as a tool to detect the decrease of bone mass. It is important to note that the demineralization protocol used does not homogeneously extract the minerals. We believe that once EDTA reaches the trabecular region in the bone epiphyses, the contact surface becomes larger in then more minerals will be extracted from that region. Therefore, a great majority of the minerals originate from the trabecular region, which may have caused a lower correlation with the AIB and FSAB parameters. We suggested that, in studies that aim to use this demineralization protocol, the demineralization should be restricted to the bone region that will be reached by the ultrasonic beam, preserving the other intact areas so that mineral loss will not be falsely associated the QUS parameters. In the real case, the bone region that presents the most significant mineral loss should be monitored by QUS. As shown by Machado *et al*.^27^, demineralization with EDTA extracts bone minerals from the most superficial layers of cortical bone. In osteoporosis and other bone metabolic pathologies, this pattern is quite different. Even so they showed a good correlation between ultrasound time-of-flight and calcium loss, indicating that mineral content plays a key role on ultrasound measurements. Indeed, the demineralization process with EDTA is far from reality. Nevertheless, our aim was to study the effect of mineral loss on QUS measurements, compared to QCT and inductively coupled plasma optical emission spectrometry measurements for validation.

The first important change occurred on the Day 3, when a decay could be observed in IRC, indicating significant demineralization on the surface of the diaphysis. This decay tendency is maintained until the Day 5, when it became stabilized. AIB increased progressively until the Day 3. A strong decay of AIB is observed on the Day 4 for all samples, coinciding with the minimum value of IRC and contrary to the expectation that AIB would continue its trend. This sudden decrease seems to indicate the existence of a calcified structure in the propagation direction (Wolff Law). On Day 5, AIB resumes its trend of increase, suggesting that EDTA has broken the aforementioned solid barrier. Further, on Day 5, no significant variation was observed in IRC and AIB compared to Day 4, which agrees with the small decay of the Ca, Mg and P curves. Monitoring the ions loss, during the demineralization process showed the importance of these elements for the acoustic and structural properties of bone.

As demineralization occurs, cortical bone becomes less dense, pores tend to enlarge, thus allowing more ultrasonic energy to penetrate inside the bone. Consequently, more energy interacts within the bone structure, augmenting the backscattering and thereby increasing AIB. This increase was observed until the Day 3 for all the samples at different proportions. The AIB decay that occurred on Day 4 for all samples suggests some anatomical structural peculiarity, perhaps a denser structure, that may be due to the mechanical tension distribution in bone (Wolff Law).

QCT provides trend measurements of bone density of the cortical bone, which are used as reference values. A positive relation between the reflection parameters (IRC and FSRTF) and bone density surface was identified. IRC showed a higher correlation coefficient than FSRTF. It is natural to suppose that, as density in the cortical region decreases, AIB and FSAB values would increase in equal proportion as more ultrasonic energy penetrates the bone structures. Nevertheless, attenuation decreases the backscattering energy. This fact may explain the lower correlation between the scattering parameters (AIB and FSAB) and QCT when compared to the correlation between the reflection parameters (IRC and FSRTF) and QCT. It should be emphasized, that the proposal of the present study to use of backscatter parameters (AIB and FSAB) contributed to better understand the behavior of the ultrasonic wave during the demineralization process, as they bring complementary information from the inside of the bone. In a pilot study^36^, we monitored change in bone density and correlation between two reflection parameters (FSIR and IRC) and BMD from 5 days of study. So, the four parameters used in the present study contributed to better understand the behavior of the ultrasonic wave during the demineralization process.

The results indicate a clear relation between demineralization (as quantified by QCT) and the corresponding decrease in the reflection parameters and increase in the scattering parameters. The trend analysis of the fall curve of the chemical elements showed a better relationship between IRC and QCT than between FSRTF and QCT, suggesting that the coincidence between QUS and QCT measurement points must be improved. However, the experiments indicate that the parameters have the potential to characterize bone.

## Methods

### Ethical issues

This research was approved by the Ethical Committee for the Use of Laboratory Animals in Research of the Faculty of Medicine of the Federal University of Rio de Janeiro (UFRJ), Brazil (Protocol N. 18/11), and followed the Guide for Care and Use of Animals in Research^37^.

#### Samples and demineralization process

Eight *in vitro* samples of fresh right femurs (3.16 ± 0.10 mm in diameter and 34.00 ± 0.30 mm in length) were obtained from eight Wistar rats (*Rattus Norvegicus Albinus*), the samples average weight was 0.54 ± 0.21 g. Rat number 5 did not have epiphysis as it was lost during the bone disarticulation procedure. Soft tissues were dissected with a surgical scalpel and scissors, and the samples were maintained for 30 days in the presence of beetle larvae (*Dermestes Maculatus*) to completely remove soft tissue and marrow^38^.

**Figure 4:**
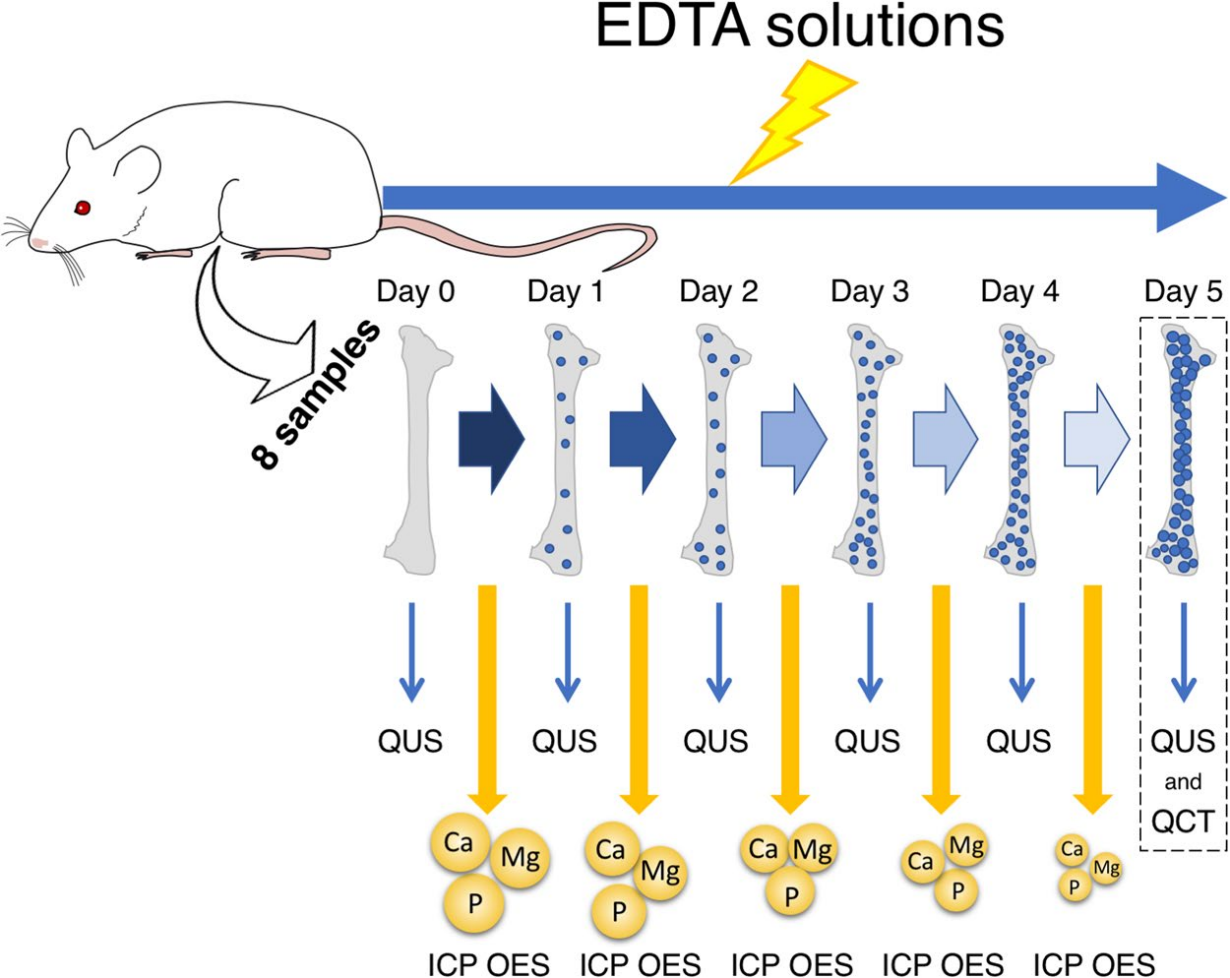
Experimental diagram. Progressive demineralization each day. EDTA - Ethylenediaminetetraacetic Acid solutions; QUS - Quantitative Ultrasound; QCT - Quantitative Computed Tomography; ICP OES - coupled plasma optical emission spectrometry. Day 0 - integer bone. Demineralization occurs from Day 1 to Day 5.

The demineralization protocol consisted of immersing the rat femur samples in a 25-mL solution of Ethylenediaminetetraacetic Acid (EDTA) disodium salt (Sigma-Aldrich®, Missouri, USA) at pH = 8 and 0.376 M concentration for 24 h at 25.0 ± 1.5 °C, and a new solution and new reservoir were used each day^27^. After each 24-h immersion, the samples were monitored by QUS and ICP OES. This procedure was followed for 6 days (Obs: Day 0 - integer bone, no EDTA immersion), and then, the samples underwent QCT measurements (Fig. 4).

### Quantitative Ultrasound (QUS) measurements

As the rat femur thickness is small (3.16 ± 0.10 mm), it was necessary to use a 5-MHz transducer for acceptable resolution (wavelength ≈ 0.6 mm in bone) and penetration^12^.

**Figure 5:**
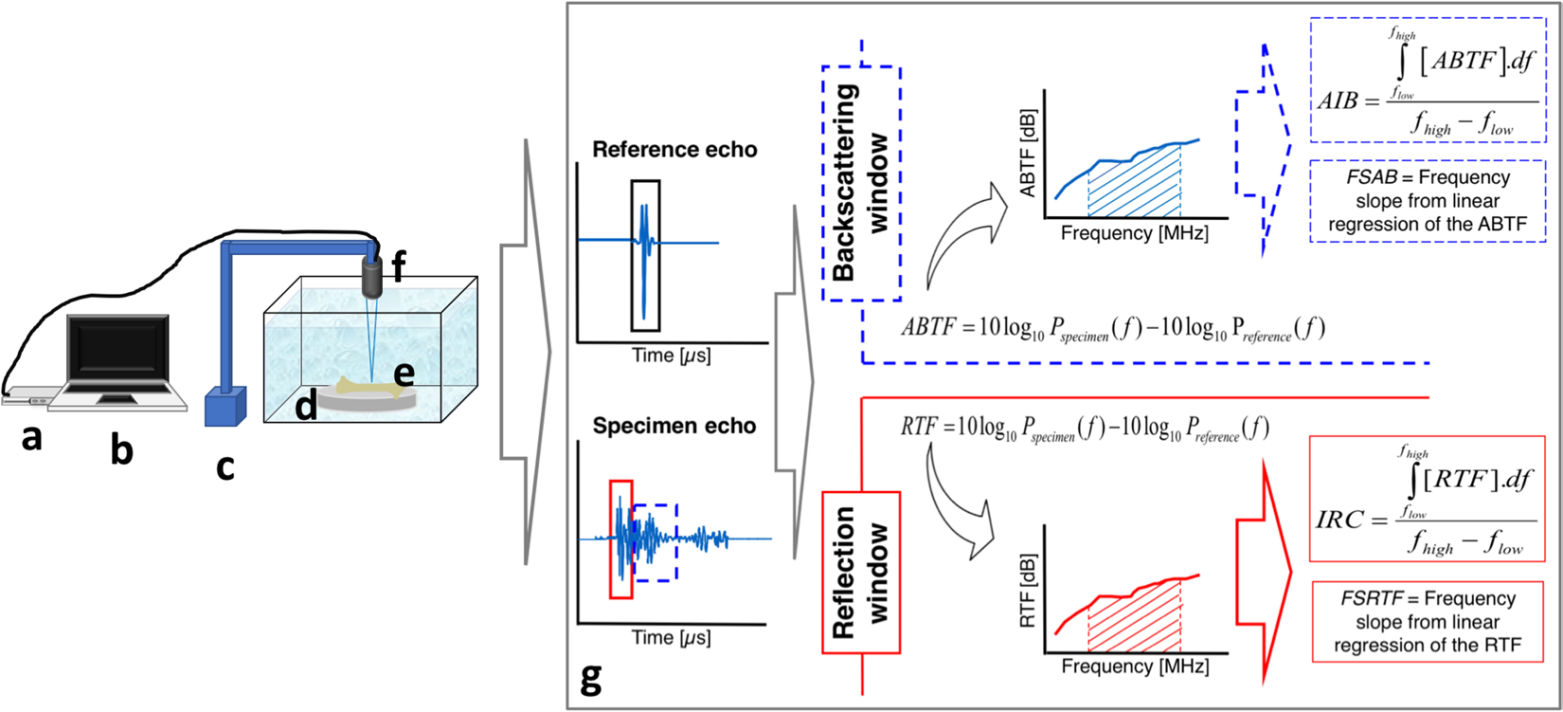
Experimental setup and signal processing scheme to obtains the QUS parameters. (**a**) US-Key Pulse Generator; (**b**) Notebook; (**c**) Stereo-static assembly; (**d**) Polished reflective steel plate; (**e**) Femur sample; (**f**) 5-MHz transducer; (**g**) Quantitative Ultrasound (QUS) measurements to estimate the ultrasonic parameters.

A 5-MHz transducer (model V326, Olympus® NDT Inc., Massachusetts, USA) with focus diameter 2.57 mm was excited by a US-key pulse generator (Gaussian pulse, 1.75 ms, 140 points sampled at 80 MHz; Lecoeur Electronique®, Loiret, FR) to insonate the samples immersed in distilled water (20.6 ± 0.6 °C) (Fig. 5).

Echo signals from the bone surface were acquired from the femur samples positioned perpendicular to the US beam at the focal length (69.3 mm). The region of interest (ROI) was at the lateral middle third of the femur diaphysis. A total of three signals were acquired from each femur with a 1.5-mm step controlled by a stereotactic holder (2-µm resolution). Reference signals were collected from a 5.80-cm-thick steel plate at the same distance.

QUS parameters were estimated from the three signals, and the average of these values was taken as representative for each sample/day.

An algorithm was developed in Matlab® (MathWorks Inc., Massachusetts, USA) to estimate the ultrasonic parameters. The radiofrequency echo segments used for bone characterization consisted of the following: *i*) the echo signal from the bone surface and *ii*) the backscattering signal from the inside of the bone.

The length of the reference echo was set by selecting the position of its extreme limits (corresponding to 10% of its maximum amplitude) to identify the bone surface echo^12^. A rectangular window was used to select a region around the reference echo (steel plate) to define the limits of the bone surface echo. The segment of 4-µs duration containing the backscattered signal from the inside of the bone began from the end of the rectangular window used for the bone surface echo.

The integrated reflection coefficient (IRC) and the Frequency Slope of Reflection Transfer Function (FSRTF) were estimated based on the reflection transfer function – RTF, which is defined as follows (eq. 1):

1 $$RTF=10lo{g}_{10}{P}_{specimen}(f)-10lo{g}_{10}{P}_{reference}(f),$$

where *P*_*specimen*_ and *P*_*reference*_ are the power spectra of the reflected signal from the sample and from the reference plate, respectively.

IRC expresses the average value of the reflection in a given frequency range (*f*_*high*_ *– f*_*low*_). Integrating RTF over frequency yields the IRC (eq. 2):

2 $$IRC=\frac{{\int }_{{f}_{low}}^{{f}_{high}}(RTF)df}{{f}_{high}-{f}_{low}}.$$

FSRTF is obtained as the slope of a linear regression of the RTF versus frequency plot, and it represents the fraction of reflection related to each frequency.

The parameters apparent integrated backscatter (AIB) and frequency slope of apparent backscatter (FSAB) are based on the apparent backscatter transfer function - *ABTF* (eq. 3), which has a definition similar to that of RTF^39^:

3 $$ABTF=10lo{g}_{10}{P}_{specimen}(f)-10lo{g}_{10}{P}_{reference}(f),$$

where *P*_*specimen*_ and *P*_*reference*_ are the power spectra of the backscattering signal from the sample and reflected signal from the reference plate, respectively.

AIB expresses the average value of the apparent backscatter in a studied frequency range and is obtained by integrating the ABTF curve.

FSAB represents the fraction of the apparent backscatter related to each frequency and is obtained as the slope of a linear regression of the ABTF versus frequency plot.

AIB and FSAB were originally defined to study backscattering from cancellous bone^39^. In the present study the idea is that the more the bone samples are decalcified, more backscattering energy comes from the inside of the bone (cancellous bone) as cortical bone is less integer. So, AIB and FSAB should be affected by that physical behavior.

### Quantitative Computed Tomography (QCT) measurements

An Optima PET/CT 560 (GE Healthcare, ©General Electric Company, Chicago, USA) camera was used. The protocol of acquisition consisted of 0.65-mm-thick axial slices, 80 kVp, and 200 mA, with a total acquisition time of 10 s. On the last day, the femurs were placed on the scanning plate, and the tomographic images were processed with Osirix Imaging Software (©Pixmeo SARL) for the bone density analysis (Hounsfield units) of the femur diaphysis. These data were used as the reference values for bone density.

### Inductively coupled plasma optical emission spectrometry measurements

Calcium (Ca), magnesium (Mg), and phosphorus (P) concentrations in the EDTA solutions (after bone immersion) were measured on an Inductively Coupled Plasma Optical Emission Spectrometer with a dual-view (axial and radial) configuration (Thermo Scientific, model iCAP 6300, Cambridge, UK), equipped with a MiraMist nebulizer (MiraMist CE, Burgener Research Inc., Ontario, Canada), cyclonic spray chamber, charge-coupled device (CCD) detector, and iTEVA 2.4 software for data acquisition. Phosphorous concentrations were determined in axial view, while Ca and Mg concentrations were determined in radial view. External calibration using matrix matching was employed for preparation of the analytical curves with six EDTA independent volume samples all with the same concentration. Analytical curves with six standard solutions were employed for instrument calibration, and quantification was performed by interpolation. Analytical solutions of Ca, Mg, and P were obtained by adequate dilution of 1000 mgL^−1^ of analytical-grade stock standard solutions (Quimlab Química&Metrologia®, Jardim Califórnia, Jacareí, São Paulo, Brazil) by using ultrapure water from a Milli-Q® system model Direct 8 (Merck Millipore, Billerica, Massachusetts, USA) containing adequate amounts of ethylenediaminetetraacetic acid disodium salt, until the desired concentrations were obtained. Owing to the high concentration of some analytes and the high content of salts (the matrix was composed of 0.376 molL^−1^ of EDTA, corresponding to approximately 140 gL^−1^), samples were diluted to 1:100 (v/v) using ultrapure water. Table 1 lists the instrumental conditions used.

**Table 1:** Instrumental parameters used for Ca, Mg, and P determination in EDTA solutions after bone immersion.

Variables	Figures
Radiofrequency power (W)	1200
Plasma gas flow rate (L min^−1^)	12
Auxiliary gas flow rate (L min^−1^)	1.00
Nebulizer gas pressure (bar)	0.19
Peristaltic pump rate (rpm)	50
Integration time (s)	1
Analysis replicate number	3
Analytical wavelength (nm)	Ca: 393.366
	Mg: 279.553
	P: 213.618

### Statistical Analysis

We arrived at the n = 8 number of experimental units by conducting a standard power calculation based on the value α = 0.05, a correlation coefficient of 0.85 (determined from the previous experiment)^36^, and a test power of 80%.

Pearson’s test was performed to verify the correlation between the concentrations of the chemical elements. To calculate the correlation coefficients between ultrasonic parameters and QCT, linear regression was performed. The linear trend of the ultrasonic parameters was analyzed as a function of bone demineralization with EDTA. A significance level of α = 0.05 and the 95% confidence interval were assumed. Data analysis was performed using Microsoft Excel® and SigmaStat Software version 3.5 (Systat Software Inc., CA, USA).

### Data availability

The data generated during this study are available from the corresponding author upon reasonable request.

### Ethical approval

All applicable international, national, and/or institutional guidelines for the care and use of animals were followed.
